# Factors influencing the adoption of health promoting behaviors in overweight pregnant women: a qualitative study

**DOI:** 10.1186/s12884-019-2199-5

**Published:** 2019-01-28

**Authors:** Azita Fathnezhad-Kazemi, Sepideh Hajian

**Affiliations:** 1 Student Research Committee, School of Nursing and Midwifery, Shahid Beheshti University of Medical Sciences, Tehran, Iran; 2 Department of Midwifery & Reproductive Health, Faculty of Nursing & Midwifery, Shahid Beheshti University of Medical Sciences, Tehran, Iran

**Keywords:** Health promotion, Pregnancy, Overweight, Qualitative study

## Abstract

**Background:**

The ability to adopt and implement health promotion behaviors is one of the most important determinants of health status. Various factors affect the successful changing of behaviors and choosing a healthy lifestyle. The present study aims at exploring the experiences of overweight pregnant women in terms of the factors influencing selection and adoption of health promoting behaviors during pregnancy.

**Methods:**

This qualitative study reports the findings of individual and group in-depth interviews with 32 overweight pregnant women using semi-structured questions which was conducted in Tabriz-Iran in 2017 and lasted for 6 months. The data collection continued until the saturation of the data. Participants were selected using purposive sampling and study inclusion criteria. Interviews were recorded and transcribed. Thereafter, content analysis was made using MAXQDA v. 10. Strength of data was verified by both participants and external control.

**Results:**

The reported effective factors led to identification of the two themes of two contextual perceived barriers and facilitators, which were classified into four main categories and nine subcategories: 1. Individual barriers (disabilities-additional needs in pregnancy and mental barriers) 2. Socio- environmental barriers (adverse effects of the environment, adverse effects of the relatives and financial pressures) 3. Individual facilitators (intrinsic incentives, abundance and individual skills) 4. Socio- environmental (social and family support, incentive environments, and raising awareness).

**Conclusion:**

The adoption of health behaviors and healthy lifestyle is under the mutual influence of individual characteristics and socio-environmental factors. What should be considered in planning and designing interventions is focused on removing barriers and strengthening facilitators, in particular by moderating social factors and taking into account individual needs and personal expectations.

## Background

A part of the complications in pregnancy are caused by unhealthy lifestyle and chronic diseases [1, 2]. Evidence suggests an increase in the prevalence of overweight in women of reproductive age and mothers who become pregnant while they are overweight [3, 4]. Usually, these people have an unhealthy lifestyle such as inactivity and poor diet quality [5–7], and these factors can put pregnancy at risk by maternal and neonatal complications, such as blood pressure, diabetes, early delivery, macrosomia and the need for hospitalization in the Neonatal Care Unit [3, 8, 9]. What is evident is that the adoption of a healthy lifestyle during pregnancy is an important determinant of the health and quality of life of the mother and infant [10, 11], and any decision about unhealthy behaviors can have a significant impact on health of the family and community [12, 13].

Health promoting behaviors and healthy lifestyle patterns are a fundamental concept and determinant of general health status [2, 14]. The importance of adopting health promoting behaviors is that these behaviors have the potential to prevent the occurrence and progress of chronic diseases and increase the control of individuals on their health status [14, 15]. Health promoting lifestyle emphasizes on life-improving behaviors such as regular exercising, eating nutritious foods, managing stress, avoiding high-risk behaviors, creating satisfying relationships with friends, and having a goal in life [2, 16, 17]. The health-related behaviors of the individual are problematic despite the much evidence about the benefits of healthy behaviors [18]. While women are seeking safe ways to spend the pregnancy period in a healthy manner as soon as they get pregnant and they are going to make positive behavioral changes, [17, 19, 20]. unfortunately, some pregnant women do not succeed in adopting healthy behaviors For example, the level of physical activity of women decreases during pregnancy despite the emphasis of studies on the beneficial effects of physical activity on the consequences of pregnancy [21]. Also, the results of studies show that not only nutritional interventions for controlling weight gain have also been ineffective, but also, in some cases, adaptation to pregnancy stress is also not successful [22]. Studies in different groups, including cardiac patients [23], children [24], physically disabled women [25], and pregnant women at the risk of gestational diabetes [26] indicate the involvement of various factors in behaviors and adherence to interventions which are related to healthy lifestyle.

Since prioritization of the health of mothers and efforts to provide the desired services is in some way a guarantee of the health of the family and the future generation, and because the establishment and plan to provide psychosocial and health services for specific and vulnerable groups, like the pregnant women, needs discovering and interpreting their experiences and views, and to clearly understand the perceptions of pregnant women as factors influencing health promotion behaviors which are keys to intervene and modify strategies for improving health behaviors, a qualitative study with an emphasis on the social context was designed to explore the experiences of overweight pregnant women in terms of the factors influencing selection and adoption of health promoting behaviors during pregnancy.

## Methods

### Study design and participants

This is a study with a content analysis approach [27], which has been conducted to discover factors affecting the various aspects of health promotion behaviors (nutrition, physical activity, spirituality, stress management, accountability, and social interactions) in Tabriz-Iran in 2017 and lasted for 6 months. Participants were selected based on inclusion criteria through purposive sampling among pregnant women referring to health care centers and pregnant women care clinics. These criteria include singleton pregnant women with an age range of 18–40, having the ability to understand and transfer concepts to a researcher, pregnancy of over 10 weeks, and having information about weight and height preconception in the women’s health records whom are defined as overweight according to their BMI (25–29.9). Exclusion criteria consisted of: any illnesses or medical health file indicating the need for special care or relative/absolute rest for the mother, as well as withdrawal and reluctance of the mother to continue participating in the study.

All individual and group interviews were conducted by the first author of the paper (A.FnK) as a faculty member and a Ph.D. student in reproductive health who has a history of participation in the classes of qualitative research methodology and the use of qualitative analysis software. She also has enough experience in the field of caring pregnant mothers. All steps for data recording and data analysis were conducted under the supervision of the author (S.H) as a Ph.D. student in reproductive health and the faculty member with several years of qualitative study.

### Data collection

After selecting the subjects through the history file of pregnant mothers according to the criteria for inclusion in the study, first, the purpose and reasons for the study were explained to each participant and, the times of the face-to-face interviews were set up as desired for the participants. Initially, five pilot interviews were conducted, which were not analyzed, but they helped shape the interview guide and how to do the study. Semi-structured questions were used to conduct interviews. These were formulated by reviewing the texts and based on the experience of the author who is responsible for qualitative studies. In individual interviews and focus groups, open and general questions were first asked to express their individual experiences. In both individual interviews and focus groups, we used the same interview guide Examples of these questions are: “What is your opinion about health?”, “What factors can help improve your health?”, “What factors can affect your attitude in changing your health behaviors?” “In your opinion, what factors force the pregnant woman to leave the healthy behaviors despite of knowing about their benefits?”, “How can these factors affect your health?” To explore the participants’ experiences, the interviews continued on the basis of their answers and questions like “What do you mean?”, “Please explain”, “Can you give us an example” were asked for a better understanding. To document the data, interviews were first recorded and then transcribed at the right time. Field notes were used as much as possible and non-verbal data such as tone and gestures was also recorded. Interviews were conducted at care centers for pregnant women or at the workplace of the participants, in an isolated room without the presence of anyone except the participant. A code and nickname were assigned to each participant. Interviews lasted from 30 min to 60 min. Interviews continued to saturate the data and until a new classification wasn’t created. Data saturation was obtained on the 14th interview. Nevertheless, two other interviews were conducted to ensure that it was completed. A total of 16 individual interviews and 3 Focus groups (two five-member interviews and a six-member interview) were conducted. And 32 people participated in the interviews.

Two of the interviews were discontinued because of the participants’ dissatisfaction with the continuation of the interview. In one case, a 19-year-old participant was excluded from the study due to she’s inability to answer the questions, and in the latter case, because of the mother’s dissatisfaction and frustration to continue the interview. One of the interviews was interrupted due to ambient noise and was repeated a few days later.

### Data analysis

Data was analyzed based on content analysis with a conventional approach [28]. The advantage of this method was to collect data from the participants directly without imposing any theoretical views by the interviewer. Data analysis was performed simultaneously with each interview MAXQDA v. 10, after recording on paper using. Identified codes were the result of semantic units of the participants’ comments.

### Rigor and trustworthiness

The interviewer tried to establish a friendly relationship with the participants to validate the results. To increase the validity of the data, the interviews were submitted to the participants after transcription in order to affirm their statements, and if necessary, additional statements were added to the data. Authors were reviewed data for several times and revised word -by-word, and the major sentences and concepts in each line or paragraph were specified and a code was assigned to every sentence. Coding was performed using the participant’s own words or based on reasoning that the researcher made according to the concepts which have been contained in the data. The transcriptions were submitted to colleague along with the extracted codes and categories, and his viewpoints were used regularly. Also, external viewpoints were used to increase the reliability; such that by provision of the initial codes which have been derived from the analysis and examples of how the concepts were extracted for the four researchers of the Reproductive health, who acted as the external observers, it was determined whether they also had a similar understanding of the data.

## Results

In this study, the mean age of the participants was equal to 29.5 (5.04), the mean age of the pregnancy was equal to 27.53 (7.55) and for most of them (20, 62.5%), it was the first pregnancy. More than two thirds of the participants had higher education degree and they were housekeepers (Table 1: Demographic characteristics of participants).

**Table 1:** Sociodemographic characteristics of participants

Variable	Mean(SD)
Pre-pregnancy BMI	27.54(1.37)
Variable	Number (Percent)
Age (in years)	
18–24	6 (18.8)
25–34	19 (59.3)
35 or above	7 (21.9)
Number of pregnancies	
1	20 (62.5)
2 or above	12 (37.5)
Gestational age in interview	
10–14	4 (12.5)
15–28	9 (28.1)
29 or above	19 (59.4)
Education	
Elementary	2 (6.3)
Secondary	5 (15.6)
High school	9 (28.1)
University	16 (50.0)
Occupation	
Housewife	23 (71.9)
Employed	7 (21.9)
Student	2 (6.2)

The codes were classified into themes and categories and sub-categories (Table 2: Examples of content analysis, coding, and categorization).

**Table 2:** An example of analysis process

Meaning unit	Code	Sub-Subcategory	Subcategory	Main Category	Theme
I spend most of my time in the office; I don’t have enough time for myself. I’m often tired when I get home from work. Sometimes I cannot even drink a glass of water in 10 h. I do not have the opportunity to get something extra (p.1)	Interference of the home chores and work	Lack of time	Mental barriers	Individual barriers	Perceived barriers
I wish I had enough time to go to gym; or, the opportunities to change my diet. I spend several hours standing, my feel swell, I don’t have the opportunity to sit down (p.1)	Lack of opportunities				
Sometimes I feel like doing something, but I’m not in the mood. I’m becoming lazy (p.2)	Lack of readiness and motivation	Lack of information and motivation			
I don’t know enough about the pregnancy exercises (p.3)	Lack of enough information				
It’s hard to get out of home, my belly is becoming bigger, its embarrass	Shame	Internal inhibitors			
I think there should not be heavy exercising, it may lead to preterm delivery (p.5)	Negative thought				
The healthcare centers told me that I am overweight and the deliver preterm. I’m fearful and worried (p.6)	Fear				
I think I’m going to miss my husband. Now he pays a lot of attention to me and loves me, I feel something will happen during the pregnancy and I will miss him (p.4)	Worrying				
Of course one may think the birth date will be premature and we have no time to arrange what we have planned for (p.7)	Anxiety				

In the process of analyzing qualitative data after categorization of the codes and eliminating similar codes, 59 codes were obtained in 19 sub-sub-categories, 9 sub-categories, 4 main categories and 2 themes of “perceived barriers” and “perceived facilitators” (Table 3: Classification of Theme).

**Table 3:** Classification of Theme, main categories and subcategories

Sub- Subcategories	Subcategories	Main categories	Theme
Physical barriers	Inabilities and additional needs in pregnancy	Individual barriers	Perceived barriers
Psychological barriers			
Lack of time	Mental barriers		
Lack of motivation and information			
Internal inhibitors			
Cultural beliefs and barriers of the community	Negative influences of the environment	socio-environmental barriers	
Insufficient facilities			
Organizational barriers			
Familial problems	Negative influences of the relatives		
Lack of knowledge of the relatives			
High life costs	Financial pressures		
Insufficient income of the family			
Internal motivation and will	Internal incentives	Personal facilitators	Perceived facilitators
Personal capabilities	Personal skills and wealth		
Having knowledge and information			
Positive influences of family and friends	Social and familial support	Socio-environmental facilitators	
Health staff			
Organizational factors	Motivational and informational environment		
Positive influences			
Providing public knowledge			

## Theme of perceived barriers

This theme consists of 2 categories, 5 sub- categories and 12 sub- sub-categories. The main categories consist of individual and socio-environmental barriers.

### Main category of individual barriers

Individual barriers include a main category of individual factors which prevent health promoting behaviors. This main category is extracted from 2 subcategories and 5 sub-sub-categories: inabilities and additional needs in pregnancy (physical inhibitors, psychological inhibitors) and mental barriers (lack of time, lack of motivation and information, internal inhibitors).

In terms of inabilities and additional needs during the pregnancy, most participants referred to the constraints which are resulting from physical and functional changes in the body organs during the pregnancy. They said:

*“During pregnancy, walking will be made tougher due to gaining weight, especially if the person is already obese. I think thin people don’t have these problems. During pregnancy, one has lower energy and gets tired soon. Anemia may also lead to lack of energy and weakness. For example, 4 to 5 months after getting pregnant, I found out that my hemoglobin count is on 10, I was very weak. I couldn’t do my house chore.” (p.7).*

*“Now (during pregnancy), I cannot drink milk. I have not drunk a glass of milk since I got pregnant. I think this is related to being pregnant. I have to consume more of healthy foods.” (p.2).*

*“I like jogging; however, I feel ache in my womb when I walk.” (p.8).*

In terms of psychological changes which prevent from taking healthcare measures, one of the mothers said:

*“At the beginning of the pregnancy period, I was a little excited and I ate a lot due to which. I liked eating. I got sensitive during pregnancy. I got angry and discomforted soon and cry a lot.” (p.1).*

The interference of the duties which have been assigned at home and at work and the loss of opportunities were the important factors that were mentioned by the working and employed women. Two *of the mothers stated:*

*“When I come back home from work, I have to do the housework and pay attention to my child and husband. So, I don’t have any time for myself. I like to exercise more, to improve my diet, but I have no time for these.” (p.1).*

*“I think, at the first pregnancy, the mother has more time for herself, but in the next pregnancy she has another baby, she does not have time for herself. Now I have to pay attention for my children also when my husband comes home, I have to pay attention to what he wants, so I don’t have enough time for myself”* Among the important barriers of taking health measures, we can mention the lack of motivation and lack of sufficient information. Pregnant women stated that:

*“Exercise is necessary for our health, but we are not used to it, we do not take it serious. We do not substitute exercise with our chores.” (p.7).*

*“I do not know about weight gain and its effects. I do not know anything I and do not ask about it.” (p.8).*

*“I do not know about the exercises that can be performed during pregnancy.” (p.3).*

Among the other personal aspects which have been mentioned by the participants, the intrinsic inhibitors such as feeling embarrassment, fear or worries and negative thoughts can be observed. Pregnant women said:

*“I wanted to ask about sexual relationship from my doctor. I liked to have information but I’m embarrassed.” (p.10).*

*“From the middle of the pregnancy onwards, the belly comes out and you have to cover it with your scarf. I have to stay in home. I can walk less than before.” (p.11).*

*“because of I have a familial relationship with my wife, and two of my brothers are deaf, for this reason I have a lot of stress that my child may have a problem.” (p.8).*

### Main category of socio-economic barriers

The socio-economic barriers include environmental, social and economic factors that make problems for the pregnant women in taking health measures. This main category is extracted from the subcategories of negative influences of environment, negative influences of relatives, and financial pressures.

Cultural believes and barriers, insufficient facilities and organizational barriers are among the environmental factors that have a negative influence on a healthy lifestyle. In terms of cultural barriers, patriarchal environment and social beliefs are the reasons of non-participant in the health programs and behaviors. Pregnant women said in this regard:

*“I like to go to the gym, but my husband is not happy with that. He says he doesn’t like it. If you (pregnant woman) want to go to the park and use exercise facilities, People look at the woman saying that she has come with her big body to exercise and people say that is not sane for the woman so you cannot exercise comfortably.” (p.12).*

*“My husband doesn’t like sexual relationship in pregnancy. He says I don’t like my child to grow up in an environment with lust. He doesn’t like it so that he rejects sexual relationship altogether.” (p.13).*

*“I don’t accept food from anyone, specifically those that I have less trust. They say the characteristics of the ones who give you food, will affect your child.” (p.14).*

Among the other barriers that were mentioned frequently, we can refer to lack of facilities and inappropriate environmental status.

*“There is no good gym in our neighborhood. I like to go to the gym but I cannot. I may have gone to the gym if it existed near us.” (p.8).*

*“I have to eat vegetables and fruits during pregnancy. Now they use chemical fertilizers to grow these foods. The fertilizers enter the body. Chickens are grown by hormone injection, so we eat less of it because it hurts.” (p.7).*

Among the organizational barriers which have been mentioned in the interviews, the planning problems, working problems for the pregnant women, lack of government services the problems of health care staff and political problems were observed. One woman said:

*“When I come for the pregnancy care, I get tired of the crowdedness. There is no planning, so I don’t like to go for the pregnancy care. In addition, the health care provider does not guide us very well. They hurry in doing their duties and measuring blood pressure. They prescribe medications and think that they do not need to guide us.” (p.7).*

The lack of government support and the existence of political problems have been mentioned as an obstacle to the desired health outcomes by working pregnant women. One of the participants stated:

*“It’s very hard for the working women, specifically for those with alternative shifts and those who have night shifts. The government has to have the necessary plans.” (p.2).*

In addition to the negative influences of the community, familial problems, lack of family support and lack of awareness among the relatives were mentioned as factors that are affecting the adoption of lifestyle and the health status. Participating women stated that:

*“The husband has an important role. I have many problems with my husband. He is an isolated man. He doesn’t like relationship with the relatives. Also he always wants me besides him (laughs) And do not let me be in touch with my family. These irritate me.” (p.1).*

*“Because I’m away from my family, I feel more needs. I like to have someone to talk to. I like them to be beside me, not for help and assistance, but for talking.” (p.5).*

About the lack of awareness of the family, one of the participants said*: “I drink pasteurized milk. My family says I have to drink fresh cow milk that is non-pasteurized, but I tell them that the cow milk has microbes even if it is boiled.” (p12).*

One of the most influential factors in the adoption of health promoting behaviors which was mentioned frequently by pregnant women was economic problems and financial pressures and inadequate income. They said:

*“The cost of screening experiments is very high. It’s very hard to pay for all that.” (p.8).*

*“If my husband had a good job, I didn’t need to work. If he could earn well, I did not need to weave carpets. If he had a good job, I could pay more attention to myself or even exercise.” (p3).*

*“There’s no job, and no job means no money. That means there’s no good diet, no exercise. These are all achieved by money. I think bad economic situation is what prevents anyone from paying attention to health.” (p.15).*

## Theme of perceived facilitators

This theme was extracted from 2 main categories, 4 subcategories and 8 sub-subcategories. The main categories included individual and socio-environmental facilitators.

### Main category of individual facilitators

The main category of individual facilitators includes personal factors which facilitate the adoption of health promoting behaviors. This main category includes 2 subcategories and 3 sub-sub-categories: internal incentives (having motivation and will), wealth and personal skills (personal abilities, knowledge and information). The most important issue about the internal incentives is having the interest, will and incentive.

About her interest on studying and learning the suitable behaviors, one of the participants said: *“I am interested in studying about the health. I read anything about it anywhere- for example on the clinic’s wall. I read any materials that the staff would give us.” (p.8).*

Another one said: “*Some women have self-confidence and do something that they want to do (about health).” (p.16)*.

What was described as a contributing factor to health behaviors is to have individual skills, especially in terms of education, financial strength, experience and the ability to plan individually. On this issue, the pregnant women said:

*“Nowadays, the women are better off they have higher education levels and most of them are working and earning income. This is helpful, because it helps them if they can get by on their own.” (p. 17).*

“*We have to try to grow. For example, for exercising, we have to start from a minimum of 5 min and gradually increase the time so that we are used to it.” (p.7).*

In interviewing with one of the participants, she pointed to the effects of awareness: *“Because of the knowledge that I have due to education (midwifery), I take pills even though I do not like them; for example, I take iodofolic, because I know we do not have iodine. I take it inevitably because of my baby.” (p.2).*

Another one said: *“It’s good to have enough information. I have passed the family planning course in the university and I know some important things.” (P. 7).*

### Main category of socio-environmental facilitators

The main category of socio-environmental facilitators includes factors which are related to family, social and environmental life. This main category was extracted from 2 sub categories and 5 sub-sub-categories included social support (support and appropriate relationship^’^s familial and health staff support) and the motivational environment and general awareness (positive environmental influence, organizational factors, public awareness).

Family and social support is one of the facilitators of adopting health promoting behaviors. In this regard, the support which had been received from the husband was emphasized by pregnant women. In addition, the support from other family members and health care providers was also mentioned as facilitators. For example, the women stated:

*“It’s true that the infant is carried by me, but my husband has to cooperate with me, he shouldn’t leave me alone and should help me.” (p.18).*

“*Being around your family and relatives, gives you confidence. We know that someone supports us, someone that we can trust in.” (p.19).*

“*I think the physiological delivery classes are better, because in these classes, we ask any questions we have. When you have information, you are calm and no anxiety will remain.” (p.20).*

Other socio-environmental facilitators are based on choosing a healthy lifestyle, availability and accessing the health facilities and environments, especially those that are specific to pregnant women. For example, several participants said:

“*The government has to consider training classes for the pregnant women during the pregnancy period; e.x., the training classes which have been held by the health centers are OK now.” (p.21).*

*“I am very happy that I could take all my pregnancy tests; it would have been costly if we were not under the coverage of insurance.” (p.21).*

*“If there is a special park for ladies, women can use the exercise facilities in the parks.” (p.21).*

Other aspects that mentioned by pregnant women, including positive effects of the living environment, such as cultural issues and motivating factors in the community, can play a positive role in adopting health promoting behaviors. One pregnant woman said:

“*I think now women can do anything they want. In the past there were constraints but this is now getting better and women are very comfortable.”* Also, she said that *“observing other people’s activity on health behaviors is an important factor”, and added: “when you see the activity of others, you become motivated to do the same.” (p.17).*

From the participants’ point of view, public awareness can be a facilitator for health behaviors, and for most of them, media and education are very important factors in raising public awareness. One of the respondents stated:

*“Education by the media is very important, and in my opinion, the media is of top priority. Media advertising is even more important than reading a book.” (P.2).*

In addition, emphasis on the importance of educating men and raising their awareness of pregnancy-related issues was mentioned by women. They said:

*“Men also need to be educated; I wish that there were places for them to be educated just like us.” (p.1).*

## Discussion

For the first time in Iran, this study examined the views and experiences of overweight pregnant women about the factors which are affecting the adoption of health promoting behaviors. The results showed that overweight pregnant women who referred to factors that could be an inhibitor or facilitator for choosing and adopting behaviors related to healthy lifestyle.

Evidence from various studies suggests that one of the strong predictors of changing positive behaviors for improving health is understanding the barriers to choose, adopt and adhere to such behaviors. According to the study by Amiri et al., personal and environmental barriers were extracted from interviews - positive understanding of status quo, studying, unwillingness, undesirable results, and low self-esteem were among the most important personal barriers and the lack of family support, culture, inadequate education, and the lack of environmental resources were among the environmental barriers to adopt a healthy lifestyle in adolescents [29]. This is in agreement with the present study. According to the findings of this study, personal factors were expressed by the participants as perceived barriers, such as physiological changes in pregnancy, insufficient opportunities and motivation, internal negative feelings such as shame, fear and anxiety. Various studies indicated the effects of pregnancy-related changes on health-related functions that are consistent with the present study [19, 30, 31]. That is, in several studies, pregnant women reported that heavy weight during pregnancy is the barrier to physical inactivity [31, 32]. In the present study, large abdomen, heavy weight and tiredness during pregnancy were mentioned as factors which have been involved in reducing physical activity. According to Weir et al., obese pregnant women reported physical and mental changes during pregnancy as individual barriers to physical inactivity [33]. Also, African American women who were participating in the study of Befort et al. stated that an increase in eating habits in pregnancy would lead to weight gain [30]. Based on the findings of other studies about pregnant women, they think that they are two people who need more food and rest [33, 34].

In this study, the lack of enough opportunities for behaviors such as physical activity, the preparation of the desired food and being with friends and recreations, were mentioned as important factors especially for the working pregnant women, and those who have children. This finding is in line with the results of other studies. Despite that insufficient time could be due to the culture of the Iranian community, in which, housekeeping and parenting is among the responsibilities of mother, which was more pronounced for the working women, lack of motivation and proper health habits were among the personal factors. One of the important factors was the lack of physical activity. In the study of Geense et al., as stated by the health care providers, lack of motivation is what leads to disregard for the healthy behaviors on the part of the women [1]. It seems that the lack of awareness of the consequences of ignorance of health behaviors is a reason for not participating in these behaviors. As noted in the study by Sue et al., gaining information about the consequences for the fetus led to an increase in motivation [22].

Connelly et al. (2015) reported that fears and worries of harming the baby were barriers to exercise for the pregnant women [35]. Based on the findings of the present study, participants discussed anxiety, fear and stress during pregnancy, which had an impact on their mental health. They requested for the psychological counseling, which is not mentioned in other studies. One of the other important factors was self-confidence, or self-efficacy, which is also mentioned in other studies.

According to other studies, lack of support or health care providers, and the high living costs are major barriers to perform healthy behaviors, which are consistent with the current study. Cultural factors, lack of facilities and the way of treating pregnant women by health care providers, family problems and lack of awareness of the relatives were the barriers to adopt healthy lifestyle behaviors. Despite that, in other studies, these things are not detailed, but some of them are mentioned. For example, in Edwards et al., one of the factors which is influencing smoking is having a smoker husband [20]. Or, in the study of Nikolopoulos et al., poor relationship with the health care providers and the inappropriate care were the contributing factors, such that care services were only for documenting the vital signs and the weight of pregnant women and, according to pregnant women, they did not receive appropriate counseling [36]. Additionally, in the study by Connelly et al., community culture can be a barrier to adopt a healthy lifestyle that is consistent with this study [35]. According to this study, the existence of patriarchal environment is a factor in not adopting healthy behaviors like participation in collective activities and outdoor activities, which has not been addressed in other studies.

Personal will, higher education, financial power and gaining information have been mentioned as the individual factors which are facilitating the adoption of health-related behaviors. According to the quantitative studies, self-efficacy and personal will have been reported as a significant predictor of health behaviors [37]. Most studies have shown a direct relationship between the level of education of pregnant women and the adoption of health promoting behaviors such as physical activity and nutrition [38–40]. However, in the study by Walker et al., employment had no effect on the level of healthy behaviors. Although researchers have not achieved significant results in this quantitative study [41], but from the viewpoint of the participants in this study, higher education, which is a result of changes in modern life, plays an important role in increasing the level of awareness, employment and financial independence of women. What is important was that pregnant women pointed to university courses regarding family planning and pregnancy methods, which, would lead to an understanding of changes in pregnancy and help in choosing healthy behaviors by increasing their information. This was not directly mentioned in other studies, which can be due to the existence of educational differences.

Family support, especially by the husband, support from health care providers and the existence of an incentive environment, including education from the media, access to health facilities and other facilities specific to pregnant women was among the socio-environmental facilitators for health promoting behaviors. Family and social support is an important factor in enabling people to choose, adopt and keep up with healthy behaviors, which is also mentioned in other studies [42]. The role of mass media in informing and receiving information from the Internet were often emphasized by women in this study. The participants in Edvardsson et al. (2011) found communication channels (face-to-face, telephone and Internet interviews) effective in changing their behaviors [19]. In the qualitative study which has been conducted by Sanchez & Jones, the Internet, mass media (for example, TV), and health service providers have been reported as sources of information for health promotion, which is in agreement with the present study [43]. It seems that by strengthening media education programs and creating valid internet sites for education, pregnant women can be supported in choosing appropriate behaviors.

According to our findings, special facilities, especially stadiums and parks which had been dedicated to pregnant women, were mentioned as facilitators of the adoption of a healthy lifestyle, especially physical activity, and this could be due to the culture of Iranian society. Also, despite that pregnant women emphasized the activities and adherence to group education classes, especially in terms of sharing information and experiences with other pregnant women, but they often sought specific advice, especially in terms of nutrition and psychology. This was not mentioned in other studies.

In general, it can be said that in order to improve the health of women, the use of individualized and aggregate strategies should be considered according to the needs of pregnant women. In this way, the best step can be women’s empowerment through increased levels of literacy and levels of participation in society for access to welfare and health. Increasing social support through training and implementing media plans should also be considered. Promotion and improving the role of men and their participation in women’s health is an important factor in this regard. Another important issue is the use of personalized counseling, in particular on the appropriate diet, physical activity, and psychological counseling for pregnant women. These strategies should be used by policy makers, planners, managers, researchers and healthcare providers to promote women’s health.

### Strengths and limitations

In this qualitative study, only the experiences and views of overweight pregnant women are included as the factors which are influencing the adoption of health promotion behaviors. It seems that appropriate interventions are needed to review the opinions and experiences of family and health care providers. Also, most participants in this study had uncomplicated pregnancies, which may affect their perceptions and experiences. In addition, women with different body mass index, such as obese and lean, may also mention different experiences.

## Conclusion

Overweight pregnant women stated their experiences regarding the factors which are affecting the adoption of health promoting behaviors as the individual and socio-environmental inhibitors and facilitators. Understanding the importance of changing behaviors is one of the determinants of adopting the health-related behaviors because pregnant women tend to change to positive behaviors because of their health concerns for their infants. For this reason, pregnancy can be a good time for adopting health measures. So, this opportunity should be used well and what should be considered in the planning and design of interventions is the focus on removing barriers and strengthening facilitators, especially by moderating social factors and taking into account individual needs and personal expectations.

## Abbreviations

BMI: Body Mass Index
